# Application of DETECTER, an evolutionary genomic tool to analyze genetic variation, to the cystic fibrosis gene family

**DOI:** 10.1186/1471-2164-7-44

**Published:** 2006-03-07

**Authors:** Eric A Gaucher, Danny W De Kee, Steven A Benner

**Affiliations:** 1 Foundation for Applied Molecular Evolution, Gainesville, FL USA; 2 Department of Chemistry, University of Florida, Gainesville, FL USA

## Abstract

**Background:**

The medical community requires computational tools that distinguish missense genetic differences having phenotypic impact within the vast number of sense mutations that do not. Tools that do this will become increasingly important for those seeking to use human genome sequence data to predict disease, make prognoses, and customize therapy to individual patients.

**Results:**

An approach, termed DETECTER, is proposed to identify sites in a protein sequence where amino acid replacements are likely to have a significant effect on phenotype, including causing genetic disease. This approach uses a model-dependent tool to estimate the normalized replacement rate at individual sites in a protein sequence, based on a history of those sites extracted from an evolutionary analysis of the corresponding protein family. This tool identifies sites that have higher-than-average, average, or lower-than-average rates of change in the lineage leading to the sequence in the population of interest. The rates are then combined with sequence data to determine the likelihoods that particular amino acids were present at individual sites in the evolutionary history of the gene family. These likelihoods are used to predict whether any specific amino acid replacements, if introduced at the site in a modern human population, would have a significant impact on fitness. The DETECTER tool is used to analyze the cystic fibrosis transmembrane conductance regulator (CFTR) gene family.

**Conclusion:**

In this system, DETECTER retrodicts amino acid replacements associated with the cystic fibrosis disease with greater accuracy than alternative approaches. While this result validates this approach for this particular family of proteins only, the approach may be applicable to the analysis of polymorphisms generally, including SNPs in a human population.

## Background

A comprehensive understanding of any system, biological or non-biological, requires that we generate models for both its structure and history. This truism applies to genomics. The last decade has shown that an understanding of history can improve, sometimes dramatically, our understanding of the relation between the structure and function in a protein family [1]. Examples of protein families that illustrate this include leptin, where a historical analysis suggested that the mouse is an imperfect model for human obesity [2], aromatase, where a historical analysis determined the physiological significance of three enzymes evidently catalyzing the "same" reaction biosynthesizing reproductive steroids in pigs [3], and angiotensin converting enzyme, where resurrection of ancestral proteins provided insight into the specificity of this protease involved in regulating blood pressure [4].

Probabilistic models for the history of a protein family can be reconstructed from the amino acid sequences of the currently extant descendents of that family. The reconstruction starts with a multiple sequence alignment that represents the evolutionary relation between individual sites in the homologous family members, and a tree that captures the familial relationships of the homologous proteins themselves [5]. Computational heuristics then infer the sequences of ancestral proteins throughout the tree, at the same time as inferring nucleotide and amino acid replacements that occurred along individual branches of the tree.

Amino acids in proteins continue to be replaced in the contemporary world. Although individuals within a population are genetically far more similar than they are different, genetic differences underlie many of the physiological differences between individuals. They are also responsible for many diseases and variable responses of different individuals to their clinical therapies.

The ability to predict which mutations cause disease, or differences in how individuals respond to standard medical protocols, will rely on detailed characterizations of mutations. For missense (non-synonymous) changes in the coding regions of genes, the descriptions include the locations of mutations on a protein, the physico-chemical properties of the amino acid replacements, rates of mutation at sites based on comparisons of homologous sequences, and probabilities of inferred ancestral amino acid states during the evolutionary history for the gene of interest. These descriptions are commonly combined within the field of molecular evolution, while only recently have they been integrated for the medical sciences.

In preparation for the accumulation of human genome mutation information from single-nucleotide polymorphism databases (SNPs), the medical community will require models that incorporate the descriptions listed above in hopes of generating accurate predictions of tolerated and non-tolerated amino acid replacements within the human population. This will be a necessary step to fully use genomics as part of predictive and personalized medicines [6-8].

The role of genetic variation in human disease is exemplified by the disease cluster known as cystic fibrosis (CF). CF causes tragic and debilitating phenotypes in the pulmonary and gastrointestinal tract of patients that it afflicts. The protein most closely associated with this cluster is the cystic fibrosis transmembrane conductance regulator (CFTR). CFTR pumps chloride ions across the cellular membranes of lung, liver, digestive and reproductive tracts, pancreas, and skin tissues, *inter alia*, maintaining the hydration of extracellular secretions.

Structurally, the CFTR protein is an ATP-Binding Cassette (ABC) transporter protein that, in humans, is a peptide 1480 residues in length (~168 kDa) encoded by a gene on chromosome 7 with 6129 nucleotides [9-11]. The protein has five domains. Two of these domains span the membrane (MSDs); each of these comprises 6 transmembrane helices that form a chloride ion channel. The CFTR also has two nucleotide-binding domains (NBDs) that bind and hydrolyze ATP, and a regulatory domain.

Missense mutations in the membrane-spanning domains of CFTR are the molecular etiology of the disease in many cystic fibrosis patients [12-17]. The Cystic Fibrosis Mutation Database collects 108 of these mutations [18]. The database does not record mutations that do not create the disease, unless multiple variations (which need not all be responsible for the disease) are present in a single diseased patient. This makes the database a valuable resource for testing new ideas to identify variation that might be the source of disease.

Here, we introduce a new approach to determine whether an amino acid replacement at a site in a protein is more or less likely to have a significant impact on fitness, including causing a disease. The model attempts to detect mutations that lead to clinical diseases regardless of the mutation's role in recessive and dominant patterns of inheritance [19]. In this manner, the approach can also identify heterozygous recessive changes, with the potential to cause disease, within a carrier background.

Our approach exploits contemporary sequence data to reconstruct the evolutionary history of the site using model-dependent mathematical heuristics. The approach then identifies sites that have historical normalized replacement rates that are higher, average, or lower than the typical site in the protein. It then infers the likelihood that any specific amino acid was present at that site over the period of history defined by the tree. Sites having lower than average historical amino acid replacement rates are hypothesized as being sites most likely to hold phenotypically significant changes in a modern population. Amino acids that have a low probability of having been present at that site during the evolutionary history are more likely to have phenotypic impact if found in the present day population. We test this approach using the CFTR protein family as a model.

## Results and discussion

**Figure 1:**
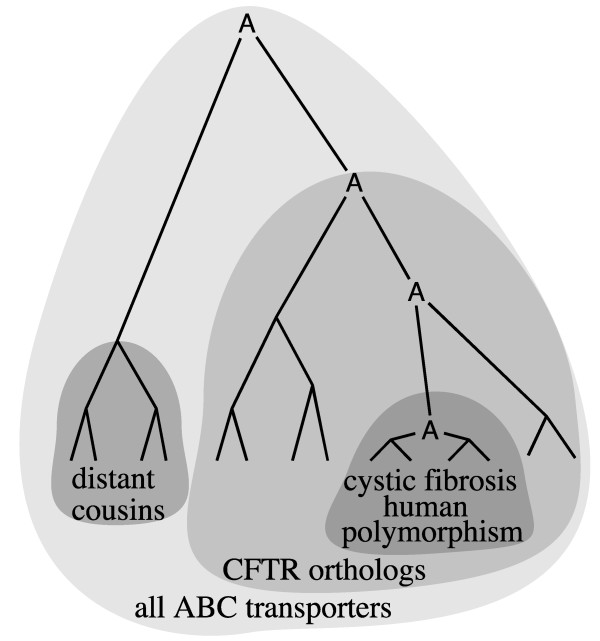
Phylogenetic schematic highlighting the relationships between ABC transporters, mammalian CFTRs, and human CFTR polymorphisms based on sequence variation.

Many ideas for predicting whether an amino acid replacement is likely to have a significant impact on the phenotype of an extant protein are based on the notion that amino acid *X *at site *i *will be tolerated in a current population if, and only if, *X *was tolerated historically in a predecessor population. A less stringent application of the same notion asks whether *X *has been tolerated in a homologous protein that is not a direct ancestor of the current population of interest (Figure 1), but is related as a "distant cousin". This notion can be made still weaker by constructing it in probabilistic form ("*X*_*i *_is more likely to be tolerated if it appears in an ancestor, or in a distant cousin"). Further, the probabilities might be parameterized depending on the amino acid replacement being considered.

This notion is both obvious and fully logical in certain cases. For example, if an active site histidine is required at position 12 in a protein and required for catalytic activity, and if the catalytic activity provided by that histidine in that protein is required to confer fitness on the host, then replacing His12 by any of the other 19 amino acids will cause a disease in the modern host. Further, the replacement will not have occurred in the past, as the mutation behind the replacement would not have been fixed in the population. Any individual having it would have lower fitness, and would not have passed that replacement on to a population of descendents.

Chemical considerations suggest that the situation must be more complex than implied by this simple model. For example, good reasons exist to suspect that whether *X*_*i *_is tolerated by a population in an extant protein depends, at least in some cases, on what amino acid is present at other sites *j*. Further, we may suspect that if multiple sites *j *are different in the cousin subfamilies, inter-site interactions, difficult to capture in any analysis might allow an amino acid to be tolerated in a cousin even though it is not tolerated at site *i *in the protein of interest [20].

Further complicating the model is the recognition that proteins are frequently recruited to have different functional phenotypes. In the example discussed above, if the catalytically active protein evolved from a protein whose role required no catalysis in an earlier period of evolutionary history, then His12 may not have been present in that period, even though its absence in a modern protein might cause disease.

### The SIFT strategy

This type of evolutionary analysis underlies a tool recently introduced by Ng and Henikoff. Known as SIFT (sorting intolerant from tolerant) [21-23], the tool constructs a profile for every site in a protein from a set of input homologous protein sequences. This profile reports a probability for each of the 20 amino acids being at that site in the generic homolog. A replacement in a contemporary population introducing amino acid *X*_*i *_is viewed as "tolerable" if that normalized probability for *X*_*i *_in the profile is greater than 0.05.

For SIFT, the input proteins can be obtained from a search using PSI-BLAST (position specific iterative-basic local alignment search tool). The cutoff to determine homologous sequences is a position-specific conservation (read, distance) metric for homologs based on classical information theory (log_2_20). Alternatively, a user can define the input dataset of homologous sequences for SIFT analysis (as done in the present study).

Recognizing the possibility that the database sequences might not carry, at any particular site, all of the amino acids that are in fact present at that site in all extant sequences on the planet, Ng and Henikoff add "pseudocounts" to the data. The number of these is based on an application of 13-component Dirichlet mixtures. Additional pseudocounts are then added based on an exponential derived from a diversity metric that includes the numerical rank (an integer from 1 to 20) for each of the amino acids at each site.

### The DETECTER strategy

It is not necessary to use such a heuristic approach to model sequence diversity in a family of proteins, especially given the availability of many empirically based tools for modeling the historical divergence of protein sequence from descendent sequences. We asked whether a tool that captures, in a more formal way, the evolutionary relationship between the input sequences might be a better means to identify phenotypically significant variation.

Under the acronym DETECTER (Determining Clinically-relevant Transmutations using Evolutionary Rationales), the tool exploits an alignment of homologous sequences, evolutionary models of DNA substitutions or amino acid replacements, phylogenetic analyses, and predictions of ancestral character states throughout the history of the gene family. These are obtained using a model-based likelihood method devised for reconstructing ancestral sequences, and implemented in PAML [24], for our test case. This method uses standard statistical theory to generate the posterior probabilities of different reconstructions given the data at a site [25-27].

For each site in the protein sequence, posterior values for all 20 amino acids are calculated and represent the probability of an given amino acid having been at that site in the protein during its evolutionary history. These values are calculated from patterns of amino acids in the alignment, models of sequence evolution, phylogenetic branch lengths, and site-specific replacement rates. Amino acid replacements having posterior probabilities greater than or equal to 0.05 are considered tolerated in the modern population, while those less than 0.05 are considered non-tolerated and may lead to aberrant protein behavior.

The tools' main differences lie in their abilities to estimate whether a site is conserved or rapidly evolving, a site's propensity to accept amino acid replacements and how much change has occurred at a site. SIFT estimates these from pseudocounts, diversity metrics based on sequence conservation, and Dirichlet mixtures, while DETECTER estimates these from the evolutionary analyses discussed above.

**Figure 2:**
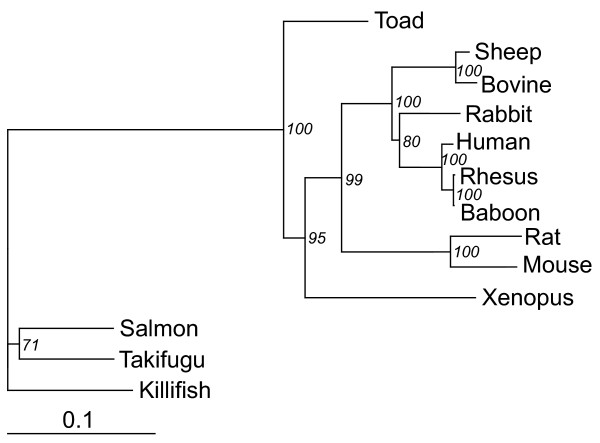
Phylogeny of the CFTR family. Bootstrap values are indicated at corresponding nodes of the tree. Scale bar represents amino acid replacements per site per unit evolutionary time.

We applied SIFT and DETECTER to the CFTR family and, separately, to related ABC transporters. These were obtained from public databases and the MasterCatalog [28]. Our analyses relied on an evolutionary tree built using the topology search tools in PAUP with complete CFTR genes [29]. The robustness of the resulting phylogeny was estimated through bootstrap analysis; it is consistent with a species tree for these organisms (Figure 2). The CFTR sequences and topology were subsequently used for a maximum likelihood analysis in the PAML phylogenetic package [30].

### Structural analysis of the CFTR mutations in the database

The disease-causing mutations in the 12 helices of the two membrane spanning domains (MSD) of CFTR have been analyzed by several groups from a structural perspective [12,15,17]. The majority (74%) of these sites in the human sequence are occupied by hydrophobic (non-polar) amino acids (FAMILYVW). Moderately polar (CPGST) and highly polar (KRENDQH) residues are found in the remaining 19% and 7% of the sites, respectively.

Schiffer-Edmundson helical wheels revealed the spatial relation of sites holding the highly polar residues in the transmembrane helices (data not shown). As has been noted in membrane spanning helices generally, polar residues are not randomly disposed around the helix. Rather, nearly half of the highly polar residues in the CFTR transmembrane regions are spaced 3–4 sites from each other, and therefore present their side chains to the same side of the helix. This suggests that these residues participate in electrostatic linkages necessary for structure/function relationships.

Of the highly polar residues in the MSDs of the native CFTR protein, arginine appears to be special. Arginine is found here at the ends of the alpha helices in which it resides. There, it may help anchor the helices [16].

**Figure 3:**
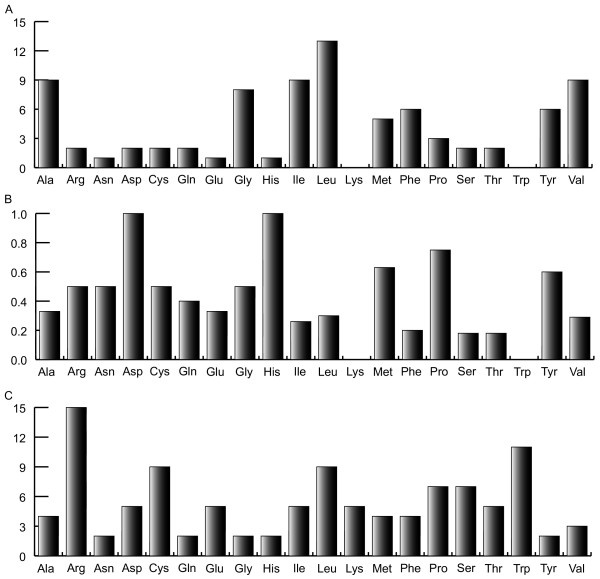
Frequencies of amino acids lost and gained through missense mutations of the CFTR protein. A, Observed frequencies of native CFTR amino acids lost through phenotypic missense mutations. B, Same as A but normalized for the particular frequencies of amino acids present in the membrane-spanning domains. C, Observed frequencies of non-native amino acids gained through phenotypic missense mutations from the CFTR mutation database.

Of the >500 disease-causing replacements in the CFTR protein from the human population, 108 are found in 83 of the 253 sites in the membrane-spanning domains (MSDs). Structural analysis shows that the majority of these replacements are found in sites that hold non-polar amino acids in the native, non-disease form of the protein. This is expected, given that most sites in the MSDs hold non-polar amino acids in the archetypal sequence. Normalizing with respect to the native frequencies of amino acids therefore gives a better understanding of the types of phenotypic changes leading to cystic fibrosis (Figure 3). The normalization shows that the loss of a polar residue (Asp, His, Pro, Gly, Arg, Asn, Cys, Gln, and Glu) has a greater chance of being associated with the disease (per polar residue) than the loss of a hydrophobic residue. This observation is consistent with both the known structural and functional importance of polar residues in transmembrane regions and previous studies that analyzed the Human Gene Mutation Database [12]. The loss of native Met and Tyr are exceptions (Figure 3b).

Providing the potential to form salt-bridge and/or hydrogen bonds within the MSDs can also be associated with the disease. The observed gain of amino acids that offer these properties support this view (Figure 3c). The most prevalent residues gained exhibit salt-bridge and H-bonding potential (Arg, Cys, Trp, Ser, Asp, Glu, Lys and Thr). The frequent gain of Pro, Leu and Ile do not support this view. Proline, however, is justified by its propensity to break helices based on its lack of backbone H-bonding potential, although other explanations may be required [31].

### Building evolutionary models for CFTR proteins

To add a historical dimension to these structural observations, we exploit an evolutionary analysis. The analysis begins with the (perhaps naïve) hypothesis that sites where replacement is likely to have a detrimental impact on fitness evolve more slowly than sites where replacement does not [32]. This suggests that one might be able to retrodict disease-causing amino acid replacements in CFTR by identifying sites that have historically evolved more slowly in the protein family.

To test this hypotheses, we needed to build an evolutionary model. Recognizing that alternative theories of evolution generate different models, we explored alternative datasets and parameters.

**Figure 4:**
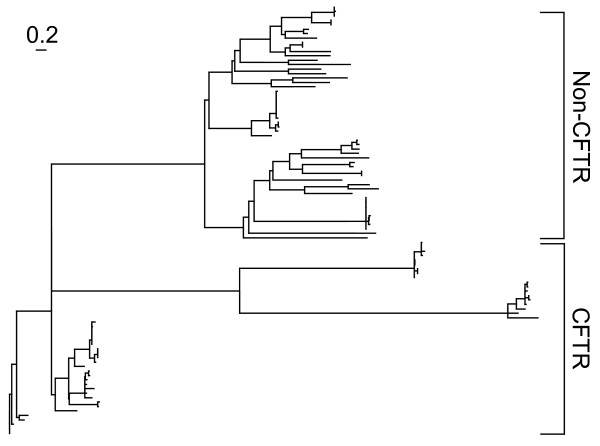
Phylogeny of the ABC-family membrane-spanning domains from PFAM.

We began by retrieving a seed multiple sequence alignment for the transmembrane regions of ABC transporters (including many not classified as CFTRs) from Pfam [33]. An amino acid replacement rate matrix for this dataset was estimated in PAML from the phylogeny shown in figure 4. The resulting transmembrane matrix (TM) was incorporated into subsequent phylogenetic analyses discussed below and compared to results obtained using a replacement matrix specific for globular proteins, Jones-Taylor-Thornton (JTT) [34].

Two datasets of CFTR sequences were subsequently constructed. The first comprised the sequences of the complete CFTR protein sequences. The second comprised only those parts of the sequences that formed the membrane spanning domains. The different replacement probabilities and trees with different branch lengths generated by these different analyses were then compared. For its ability to infer branch lengths and ancestral states, the JTT matrix (not specifically designed for membrane-embedded protein sequences) is expected to outperform the TM matrix for the complete CFTR dataset, as only 253 sites out of the ca. 1450 total sites are in contact with the lipid bilayer. Thus, a majority (roughly 83%) of the sites in the CFTR protein are expected to evolve like most globular proteins, and therefore have their amino acid replacement best represented by the JTT matrix. Consistent with this expectation, likelihood scores for the evolutionary models for the complete CFTR dataset were 13825.91 and 14740.58 for the JTT and TM matrices, respectively.

Alternatively, the TM matrix is expected to outperform the JTT matrix for the MSD-only dataset, in part because the TM matrix was based on these membrane spanning domains. The likelihood scores for these datasets were 2122.61 and 2019.63 for the JTT and TM matrices, respectively. A second-order Akaike Information Criterion (AIC_c_) test fitting the two matrices to the data supported this expectation (ΔAIC_c _= 205.9592).

### Testing the hypothesis that polymorphisms in slowly evolving sites are more likely to be associated with disease

To estimate the historical rates of replacement, a tool implemented by Yang within the PAML package (v3.14) was used. We exploited this tool's ability to examine an entire protein sequence family and generate, for each site, a normalized replacement rate based on the posterior mean probabilities of the site's extant and historical amino acid patterns residing in the individual categories of the gamma distribution. These numbers indicate the rate of replacement at the site throughout the history of the family, normalized so that the average replacement rate is unity. Thus, no site can have a normalized replacement rate below 0, but sites can (in principal) be substantially above unity. In the CFTR protein, the highest normalized replacement rate is ca. 2.8 replacements/site/unit evolutionary time.

Once normalized replacement rates were calculated for each site, we identified sites that have had higher-than-average, average, and lower than average replacement rates in the history of the membrane-spanning domains of CFTR. Based on the hypothesis, we expected that sites having lower-than-average historical replacement rates to be more likely (than the average site) to hold polymorphisms in human populations associated with CF. Conversely, we expected that sites having higher than average historical replacement rates to be less likely to hold polymorphisms associated with CF.

**Figure 5:**
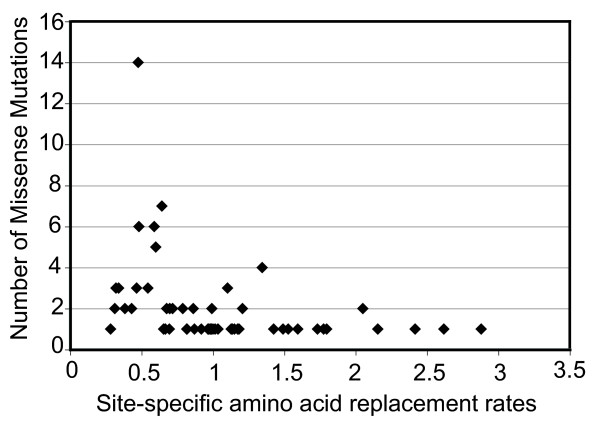
Correlation of the number of missense mutations versus the estimated site-specific amino acid replacement rate hosting the mutations.

This proved to be the case. Only 42% of the sites (106 sites) have a historical replacement rate greater than unity. In contrast, 58% of the sites (147 sites) have a historical replacement rate less than unity. Of the 108 phenotypically significant mutations in the database, however, 74% are in sites that have a below average historical replacement rate (Figure 5).

The correlation was supported more strongly by distributing the sites into six bins based on their normalized replacement rates (Table 1), and noting that those in the bin with the lowest normalized replacement rates (normalized replacement rate 0.0–0.5, 77% of the sites here host a CF mutation) were more likely to be associated with the disease than those in the next higher bin (0.5–1.0, 44% host a CF mutation), and that this trend continued to the highest bin (2.0–2.5, 17%, chi-square = 22.1 probability ≈ 0.001).

**Figure 6:**
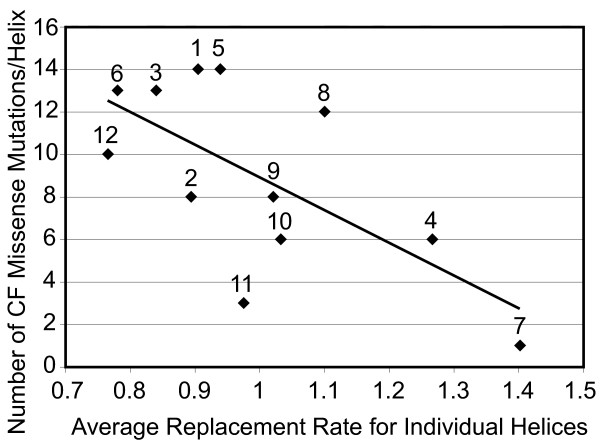
Correlation of the number of missense mutations versus the average replacement rate for the individual alpha helical transmembrane region hosting the corresponding mutations. Transmembrane helices 1–12 are numbered.

The predictive power using evolutionary rates can be extended to the 12 individual transmembrane helices of the CFTR protein. The correlation of the number of phenotypic missense mutations per individual helix to the average replacement rate of that helix highlights patterns not seen in the absence of evolutionary analyses (Figure 6). Specifically, the helices hosting a greater proportion of slowly evolving sites are more likely to give rise to phenotypic missense mutations. Supporting this view, the six slowest evolving helices host a total of 72 CF missense mutations (67%), while the six fastest evolving helices host only 36 missense mutations (33%). Further, since five of the six slowest evolving helices reside in MSD 1, mutations in this membrane spanning domain appear to be more deleterious than mutations in MSD 2.

### Extending the analysis to include the history of amino acid replacements

We then enhanced the analysis by considering the specific amino acids involved, including those inferred at individual sites throughout the evolutionary history of the CFTR family. The CFTR topology and the TM matrix were used to estimate ancestral character states at all the internal nodes of the phylogeny (Figures 2, 4). The likelihoods associated with any amino acids having been present during the history of CFTR were collected for each position of the multiple sequence alignment. Any amino acid residue having a posterior probability greater than 0.05 at any internal node of the tree for a given site was then predicted to be tolerated in the modern protein. This cutoff is arbitrary, but was chosen to be consistent with the cutoff used by Ng and Henikoff.

In drawing inferences about ancestral sequences, it is also important to be selective about what extant homologs to include in the analysis. As noted above, subfamilies within a large family of homologous proteins need not have the same "functions", but might play very different roles as a consequence of recruitment in the historical past. As has been discussed by many, subfamilies within a family recruited to perform different functions divergently evolve with different patterns of sequence evolution. In particular, there is no reason to expect that sites that have high replacement rates in one subfamily are the same as sites that have high rates of replacement in another [35], or that the patterns of replacement in an ancestral population where the function was different from the function in the modern family will accurately identify phenotypically significant variation in the modern family having the derived function.

To recognize these realities, the DETECTER tool was applied to various datasets chosen to deliberately include, or deliberately exclude, subfamilies within the ABC transporter family that had roles different from the role played by CFTRs. Separately considered were: MSD domains for CFTR-only [this same dataset was analyzed by DETECTER (D1) and SIFT] and ABC transporters [analyzed using DETECTER only (D2)].

Table 2 outlines the predictions of tolerated amino acid replacements made by DETECTER (D1 and D2) and SIFT. These were compared to the amino acid replacements associated with the CF disease in the Cystic Fibrosis Mutation Database.

The approaches performed similarly, but their differences are noteworthy. Of the 108 known mutations in the CFTR membrane spanning domains, DETECTER incorrectly predicted 8 of these mutations to be tolerated, when its construction of the history of the site was based on the D1 dataset. These constitute error, as these 8 are believed to cause disease in the human population. Thus, the DETECTER approach created ~8% false negatives. The coefficient between the replacement rate at any individual site versus the number of predicted tolerated amino acids highlighted a positive correlation (Pearson r = 0.85).

The SIFT approach, however, generated more false negatives when analyzing the same MSD-only dataset. This analysis predicted that 15 of the 108 mutations that are associated with the CF disease would be tolerated.

Last, the DETECTER tool applied to the ABC transporter dataset (D2) generated the largest number of false negatives. After considering the evolutionary history of a dataset that included ABC transporters that had functionally diverged from the CFTR role, the DETECTER tool mispredicted that 47 of the 108 mutations in the MSDs would be tolerated.

These results show the value of a historical analysis of protein sequence variation and an associated disease, cystic fibrosis. Further, they show the hazards of applying a historical analysis naively, across a family of proteins where the physiological role has itself diverged.

Some of the differences in outcome can be directly attributed to the incorporation, in a historical analysis, of proteins that do *not *play the same physiological role as the protein of interest (here, CFTRs).

Thus, the D1 and D2 analyses differ in that the former narrowly includes only those proteins that serve as CFTRs, while the latter includes ABC orthologs that do not. This is undoubtedly the cause of the large number of false negatives that arise when the DETECTER tool is applied to the ABC transporters as a whole, and the very few of false negatives that arise when the DETECTER tool is applied to CFTRs only.

Differences in the outcomes between SIFT and DETECTER (D1 dataset) reported above are not explained in this way. For example, SIFT incorrectly predicts that replacements at positions 209 (A→S), 1006 (A→E), and 1148 (N→K) will be tolerated. In fact, each of these is associated with the CF disease, and none are incorrectly predicted to be tolerated by the DETECTER tool applied to either of the datasets. Two of these replacements involve apolar-to-polar changes, while the other is polar-to-polar. The site-specific replacement rates of these positions alone (2.42, 2.62, and 0.87, respectively) do not offer much insight.

Interestingly, while the use of different amino acid replacement matrices (TM and JTT) by DETECTER had moderate effects on the overall probabilities of the inferred ancestral character states, in no instance did this affect the overall predicted tolerability of any amino acid replacement (data not shown).

All eight amino acid replacements incorrectly predicted by analysis using the DETECTER tool applied in D1 are among the 15 incorrect predictions made by SIFT. Four of these involved apolar-to-apolar replacements (I→V twice, M→I, and V→I), three moderately polar to apolar replacements (P→L twice, and S→L) and one moderately polar to highly polar replacement (G→R). Previous studies have classified this last replacement as having a high 'phenotypic propensity' for disease [14,16], and in fact occurs four times in the Cystic Fibrosis Mutation Database. Both the DETECTER and SIFT approaches incorrectly predict the G→R replacement to be tolerated at position 219 because the close homolog *Xenopus *has an Arg at this position. The tolerance of Arg at this position in *Xenopus *may be due to altered selective constraints or altered structural bonding patterns acquired to compensate for the loss of Gly at the site in this species.

Such altered selective constraints may also provide an explanation for some of the false-negatives inferred by DETECTER. Here, 4 out of the 8 sites represent cases in which the residue at the site is conserved for the mammalian sequences, and conserved-but-different for the non-mammalian sequences. Conserved-but-different patterns are often invoked to explain functional divergence between biomolecules [36]. Further, these four sites reside in helices that host the majority of CF missense mutations and have slower than average replacement rates (Figure 6). Thus, while amino acid replacements were tolerated during the divergence of mammalian and non-mammalian species, further replacements appear to be non-tolerated within any single subclade. Here, the combination of output from DETECTER and evolutionary rates provides additional information to draw conclusions on the tolerance of mutations.

Alternatively, the other half of the 8 incorrect predictions generated by DETECTER are amino acid replacements present in CF patients that also host other mutations implicated in causing the disease. These amino acid replacements may thus represent neutral polymorphisms carried within the disease background. As such, some of the apparent false predictions by DETECTER may in fact be true negatives.

## Conclusion

While statistical analyses targeted against genomic sequence databases are important in developing validated tools for use in bioinformatics, many of the most important concepts that have driven the field have emerged through the analysis of individual cases [37-39]. This is not surprising, given that proteins are organic molecules. Understanding in organic chemistry has nearly always come through the development of narratives based on case studies, where the concepts in those narratives have then been tested, modified, and expanded through the addition of further narratives. As with structure-function relations in organic molecules, structure-function analysis in proteins asks how changes in the arrangement of atoms in a protein changes its properties.

Examination of a single dataset for a single protein family does, however, have certain disadvantages. Most obviously, the approach is validated for that family only. Further, there is the risk that this family is peculiar with respect to families generally, and approaches that work here will not work generally.

These concerns aside, it is clear that adding evolutionary information to the structural information in the cystic fibrosis family provides new insights. Seventy (65%) of the 108 phenotypic missense mutations residing in the membrane-spanning domains of CFTR involve inter-class switches between apolar, moderately-, and highly-polar residues. Due to the high proportion of hydrophobic residues in the MDS, it was not surprising that phenotypic missense mutations of native apolar residues were responsible for the majority of mutations leading to cystic fibrosis.

Loss of native highly polar residues through phenotypic mutations, however, represented the largest proportion of mutations as a percentage of class. There were 17 phenotypic missense mutations associated with the 18 native highly polar residues located within the MSDs. This indicates that the physico-chemical properties of apolar residues provide specific and necessary structural and functional hydrogen bonding interactions in the MSDs.

Along similar lines, the observation that apolar-to-polar amino acid replacements comprised the largest observed number of inter- or intra-class missense mutations is consistent with the role of hydrogen bonding patterns in the membrane-spanning domains. Here, the addition of H-bonds can result in undesired interhelical crosslinks, disruption of active and/or regulatory sites, and modified helical packing through steric hindrances [14,16,17]. Forty-two of the 108 missense mutations involve apolar-to-polar replacements.

The DETECTER and SIFT approaches generate comparable predictions regarding the tolerability of phenotypic missense mutations in the CFTR protein, i.e., differentiating true negative (correctly-predicted tolerated) from false-negative (incorrectly-predicted tolerated) amino acid replacements. Notable exceptions, however, are apparent and most likely explained by the different approaches of the two programs. DETECTER relies on phylogenetic analyses and invokes models of sequence evolution tailored for specific gene families, while SIFT relies on its ability to capture models of sequence evolution indirectly through sequence alignments only. Thus, SIFT generates pseudocounts from a Dirichlet mixture to estimate expected (unobserved) sequence diversity, whereas DETECTER attempts to capture this information through branch lengths and implicit models of sequence evolution using phylogenetic analysis.

We have demonstrated the importance of capturing features of an amino acid replacement matrix (e.g., apolar-to-polar changes), site-specific evolutionary rates (validating the notion that changes in slowly evolving sites care correlated with disease states), homologous sequence divergence (close relatives versus distant cousins) for the DETECTER approach to predict the consequences of polymorphisms in the coding regions of CFTRs in the human population. While none of these analyses are unique when considered alone, their combination is unique and may represents an important contribution to clinical diagnostics.

Additional studies are required to differentiate the abilities of DETECTER and SIFT to discriminate true-positive (correctly predicted to be non-tolerated) from false-positive (incorrectly predicted to be non-tolerated) amino acid replacements for CFTR. Advances in technology enabling the collection of large amounts of SNP data will undoubtedly allow these studies to be performed in the near future, and allow the comparison of different methodologies such as DETECTER, SIFT, and POLYPHEN along these lines [23,40].

It is noteworthy that SIFT and POLYPHEN have analyzed data from other mutation databases such as the human non-synonymous single nucleotide polymorphism database [22,40,41]. Although previous studies using SIFT have demonstrated its ability to outperform analyses attempting to predict tolerated amino acid replacements based on scoring matrices alone, these studies highlight the need for algorithmic development to improve accurate predictions of non-tolerated (deleterious) amino acid replacements [21,22,42]. Additional studies will also be required to understand how sequence sample- and population-sizes affect the predictions of tolerated and non-tolerated amino acid replacements.

We have shown that incorporating models of molecular evolution to generate statements about tolerability of missense mutations can enhance the power of predictive medicine. These statements are even more powerful when correlated with known three-dimensional structural information [2,3,35,40,41,43-46]. For this reason, we expect that the structure of CFTR will provide added value to such analyses. The genomic medicine of the future will require both reliable predictions about which types of mutations cause disease (predictive medicine) and detailed understandings of the variation in different human subpopulation's responses to therapeutics (personalized medicine) [6-8].

## Methods

### Sequence data

Complete CFTR genes were collected from the Genbank database and aligned: *Homo sapiens*, human (gi:1809238); *Macaca mulatta*, rhesus (gi:3047171); *Papio anubis*, baboon (gi:5679281); *Oryctolagus cuniculus*, rabbit (gi:7442654); *Ovis aries*, sheep (gi:2506121); *Bos taurus*, bovine (gi:461719); *Rattus norvegicus*, rat (gi:34854998); *Mus musculus*, mouse (gi:20141218); *Xenopus laevis*, frog (gi:1617482); *Bufo bufo*, toad (gi:12963887); *Salmo salar*, salmon (gi:12746235); *Takifugu rubripes*, blowfish (gi:38322733); and *Fundulus heteroclitus*, killifish (gi:3015540). Additional CFTR sequences have been deposited in Genbank since we initiated our studies [47]. These sequences are not expected to affect our evolutionary analyses since we calculated relative rates opposed to absolute rates.

ABC transporter homologs of CFTR were identified by a BLAST [48] search using default parameters. Ninety seven full length sequences were retrieved and aligned. Multiple sequence alignments (MSA) were conducted by ClustalW [49], with minor adjustments made by hand.

### Amino acid replacement matrix

The ABC transporter membrane spanning domain was retrieved from the PFAM [33] database (PF00664). The multiple sequence alignment corresponding to the 'seed' was cropped from 73 to 62 sequences due to inappropriate gapping. This multiple sequence alignment was used to estimate the transmembrane rate matrix (TM) using PAML [30].

#### Akaike Information Criterion (AIC)

An AIC statistical test was invoked to determine the fit of the two un-nested amino acid replacement matrices TM and JTT to the membrane spanning domain dataset. A second-order AIC was implemented because of the small ratio of sample size to free parameters in the phylogenetic analysis (AIC_c_) [50,51]:

AICc=(−2l+2K)+2K(K+1)n−K−1
 MathType@MTEF@5@5@+=feaafiart1ev1aaatCvAUfKttLearuWrP9MDH5MBPbIqV92AaeXatLxBI9gBaebbnrfifHhDYfgasaacH8akY=wiFfYdH8Gipec8Eeeu0xXdbba9frFj0=OqFfea0dXdd9vqai=hGuQ8kuc9pgc9s8qqaq=dirpe0xb9q8qiLsFr0=vr0=vr0dc8meaabaqaciaacaGaaeqabaqabeGadaaakeaacqWGbbqqcqWGjbqscqWGdbWqdaWgaaWcbaGaem4yamgabeaakiabg2da9iabcIcaOiabgkHiTiabikdaYiabdYgaSjabgUcaRiabikdaYiabdUealjabcMcaPiabgUcaRmaalaaabaGaeGOmaiJaem4saSKaeiikaGIaem4saSKaey4kaSIaeGymaeJaeiykaKcabaGaemOBa4MaeyOeI0Iaem4saSKaeyOeI0IaeGymaedaaaaa@4745@

where *l *is the maximized log-likelihood, *K *is the number of free parameters (25 free-parameter branch estimates plus one free-parameter for the gamma distribution), and *n *is the sequence length from the multiple sequence alignment (253).

### Phylogenetic analyses

The CFTR tree topology searches were conducted using the minimum evolution criterion with 10,000 replicates of random sequence addition in PAUP [29]. Bootstrap analysis consisted of a fast-heuristic search of 1000 replicates with re-sampling.

The ABC full-length and membrane-spanning domain-only datasets were analyzed as above, with the exception of 10 and 100 replicates, respectively.

The resulting topologies estimated in PAUP were subsequently used for maximum likelihood analyses in PAML (v3.14) [30]. These included incorporating different amino acid replacement matrices (Jones-Taylor-Thornton and the estimated TM), calculating site-specific amino acid replacement rates using posterior mean rates, and estimating ancestral amino acid character states. All analyses accounted for site-specific rate variation using a discrete gamma distribution with eight rate categories.

### SIFT analyses

We defined the homologous CFTR sequences, and their subsequent alignment, for the SIFT analysis of the membrane-spanning domains. This analysis did not exploit SIFT'S ability to perform database searches to identify homologous sequences. In this way, identical datasets were used in the SIFT and DETECTER D1 analyses.

### DETECTER tool

To extract information from a PAML output (rst files) presenting the posterior probabilities of ancestral character states throughout the evolutionary history of a protein family, a Perl script was developed and is freely available for download from our server [52].

### Cystic fibrosis mutations

All mutations of the cystic fibrosis gene were downloaded from the Cystic Fibrosis Mutation Database [18]. Missense mutations residing within the 12 transmembrane helices were extracted.

## Authors' contributions

EAG conceived and developed the DETECTER tool, and prepared the manuscript. DWD developed computational tools. SAB prepared the manuscript.

## Figures and Tables

**Table 1:** Distribution of CF-causing mutations and the individual amino acid replacement rates of the sites hosting these mutations.

Normalized replacement rate	Sites	%of sites	Sites with phenotypically significant mutations (O)	Expected sites if randomly distributed (E)	(E-O)^2^/E
0.0–0.5	44	17	34	18	14.22
0.5–1.0	103	41	45	44	0.02
1.0–1.5	59	23	19	25	1.44
1.5–2.0	25	10	5	11	8.7
2.0–2.5	18	7	3	8	3.1
> 2.5	4	2	2	2	0
	253	100	108	108	27.48

**Table 2:** Comparing tolerated amino acid replacement predictions using the DETECTER and SIFT approaches to the Cystic Fibrosis Mutation Database

Site	Residues	SIFT	D1	D2	Site	Residues	SIFT	D1	D2
85	E,V			X(V)	336	K			
87	L	X	X	X	338	I			X
88	S				340	N			
89	C				346	P			
90	S			X	347	C,H,L,P			
91	R				348	K			
92	K				349	V			X
96	E			X	866	Y			
98	P,R			T(R)	912	L	X	X	X
99	L			X	913	C			
101	S			X	917	D,C			
102	P				918	M			
119	V	X	X	X	919	C			
120	T,V			X(T,V)	920	L,M	X(L)		X(L)
125	T				922	L	X		X
126	D			X	924	N			
132	M				927	P			
137	H,R				932	S			X
198	P				991	V	X	X	X
199	Y,R,Q			X(Y)	993	Y			
200	I			X	994	C			
201	M				997	F			X
205	S,R			X(S)	1005	R			
206	W,F				1006	E	X		
209	S	X			1008	D			
210	P				1009	T			X
213	V			X	1014	C			
225	R				1021	A,S			X(A,S)
227	R				1028	R,I	X(I)	X(I)	X(I)
232	D				1032	C			
237	E			X	1105	R			
239	R	X	X	X	1118	C,F			X(F)
241	R				1129	G			X
309	T,D,G,V			X(T,G,V)	1130	A			X
311	L			X	1136	T			X
314	R,E,V			X(V)	1137	V,R			X(V)
316	L	X	X	X	1139	V			X
317	A			X	1140	K			
320	V,F	X(V,F)		X(V,F)	1142	I			X
322	A			X	1147	I	X	X	X
327	R				1148	K	X		
334	W,L,Q								

D1 and D2 refer to CFTR MSD-only and ABC-homolog sequence analyses using the DETECTER approach. SIFT was applied to same dataset as in the D1 analysis.

Position numbers correspond to the human CFTR protein.

Residues listed are extracted from the Cystic Fibrosis Mutation Database.

X indicates that the predicted tolerated amino acid residue is found in the Cystic Fibrosis Mutation Database. Residues in parentheses indicate which predicted tolerated amino acid is referred to when a site hosts multiple missense mutations.
